# Executive functions and school achievement: The mediating role of learning‐related behaviour in primary school children

**DOI:** 10.1111/bjep.70053

**Published:** 2025-12-22

**Authors:** Carlotta Rivella, Paola Viterbori

**Affiliations:** ^1^ Department of Education Sciences University of Genoa Genoa Italy

**Keywords:** executive functions, learning‐related behaviours, school achievements, self‐regulated learning

## Abstract

**Background:**

Executive functions (EFs) are critical in school and closely linked to academic achievement and learning‐related behaviours (LRBs). LRBs encompass the ability to adapt to school demands, including concentration, adherence to rules, and autonomy.

**Aims:**

This correlational study aimed to examine the mediating role of LRB in the association between EF and academic achievement in the first year of primary school.

**Sample and Method:**

Ninety‐five first graders underwent a performance‐based EF assessment, involving tasks related to working memory and inhibition. Concurrently, they completed literacy and math tasks, while their teacher provided reports on their LRBs.

**Results:**

The results indicate that depending on the specific EF component or learning domain considered, diverse patterns emerge. Nevertheless, the findings consistently show that EFs are directly and indirectly associated with learning outcomes, with the mediating influence of LRBs.

**Conclusions:**

These results emphasize the importance of supporting EF development into early education curricula.

## INTRODUCTION

It is widely recognized that achievement gaps emerge early in children's educational careers and are already evident in the transition from preschool to primary school (Blair, 2002; Erer et al., 2025). Within the vast literature investigating individual differences in academic achievement, self‐regulation emerges as one of the most significant predictors of school performance (Duncan et al., 2007). Within this framework, two different bodies of research can be recognized in early achievement. One refers to the ‘executive function’ framework (Miyake et al., 2000), and the second to the ‘learning‐related behaviours’ framework (McDermott et al., 2014).

Executive function (EF) refers to a variety of different and partially overlapping cognitive skills implicated in goal‐directed behaviour (Miyake et al., 2000). These skills include inhibition, which is the capacity to suppress inappropriate responses or behaviours and to control interferences of non‐relevant stimuli; updating in working memory (WM), which is the active manipulation of the information temporarily maintained in memory; and shifting (or cognitive flexibility), which is the ability to change mental strategies, responses, or activities according to different rules or objectives. EFs are crucial as they allow the voluntary control of cognitive processes and behaviour when automatized or impulsive responses are not adequate (Pennington & Ozonoff, 1996). These abilities develop until late adolescence, with a peak during the first 5 years of life, during which EFs evolve from an undifferentiated to a multicomponential factor (Garon et al., 2008; Wiebe et al., 2011). From the age of five onward, two distinct dimensions, namely, WM and inhibition, are recognized (Miller et al., 2012; Usai et al., 2014), while cognitive flexibility emerges later (Lehto et al., 2003).

Learning‐related behaviours (LRBs) refer to a set of behaviours that facilitate learning, including active participation in class activities (Brock et al., 2009), demonstrating enthusiasm and curiosity for novel activities, persisting with learning tasks despite challenges, focusing on lessons despite feeling bad or discouraged (McDermott et al., 2014), adhering to classroom rules and displaying independence and cooperation (Sung & Wickrama, 2018). These behaviours reflect how children apply self‐regulatory skills in classroom settings and are closely linked to executive functions (EFs), which provide the cognitive foundation for goal‐directed behaviour (Brock et al., 2009; McDermott et al., 2014).

In recent years, several empirical research studies directly linked these concepts to each other (McDermott et al., 2014). Altogether, there is considerable support for the hypothesis that LRBs may be an important mediator in explaining the association between EF and academic achievement (Nesbitt et al., 2015; Sasser et al., 2015). However, despite increasing interest in this topic, some aspects remain understudied in the existing literature. First, the potential of this pathway has not yet been adequately explored in first graders, a developmental period in which children face major regulatory and cognitive demands associated with the start of formal schooling. Second, most studies have analysed EF as a single construct, while only a few have examined the specific contribution of distinct EF components to different domains of achievement. The present study aims to address these gaps. Specifically, we (1) examine whether LRBs mediate the relationship between EF and academic achievement measured concurrently in first‐grade students, and (2) explore the distinct role of individual EF components – inhibition, updating in WM, and cognitive flexibility – in predicting early achievement in literacy and mathematics.

The first year of school is an important period to study how EFs and LRBs contribute to academic success (Cortés Pascual et al., 2019). This is particularly relevant in countries where preschool curricula do not prioritize the development of school skills like reading and writing. In Italy, for instance, preschool involves children aged 3–6 years and is designed to foster autonomy, identity, and skill development, as outlined by National Guidelines (Ministry of Education, University and Research, 2018). These objectives are achieved through play, exploration, movement, manipulation, and observation. Consequently, there is significant emphasis on play and practical activities, while pre‐reading and writing activities are limited and brief. To note, preschool attendance is highly widespread in Italy, with ~ 94% of children enrolled (Italian National Institute of Statistics [Istat], 2024). When children begin elementary school, they are required to make a substantial change in terms of regulation skills and EFs (Kim et al., 2021; Morrison et al., 2019). The first year of primary school focuses on the acquisition of foundational literacy and numeracy skills, such as understanding the correspondence between phonemes and graphemes, reading and writing short sentences, and learning to read and order numbers, as well as perform simple arithmetic operations (Ministry of Education, University and Research, 2012). Here, children are required to sit for several hours a day paying attention to teachers, while hands‐on experiences and playtime are greatly reduced. A recent systematic review highlighted that EF and self‐regulated learning (SRL) are closely related during academic transition phases, such as the shift from preschool to primary school (Dörrenbächer‐Ulrich et al., 2024). The authors emphasized that early EF may serve as a foundation for the later development of self‐regulatory and metacognitive skills crucial for school success and well‐being. A better understanding of the role of these processes in school achievement at the beginning of primary school can provide educators with guidance on how to support their students' achievement and prevent or reduce disparities, together with guidance on which dimensions are important to support in early years to promote later school success.

### Executive functions and school achievement

In the school context, EF skills are particularly useful, as schools require children to focus and maintain attention on tasks, even in the presence of distracting stimuli, remember and follow rules and instructions, and inhibit inappropriate behaviours (Morrison et al., 2010). As such, EF constitutes a critical element of school readiness (Blair, 2002).

The direct impact of EFs on school achievement has been a focal point of research, revealing a robust association with learning abilities in both mathematical and literacy domains across various developmental stages (Bull & Lee, 2014). For instance, mental calculation requires children to manipulate and concurrently hold information in mind, while other math tasks, like problem‐solving, demand inhibitory abilities to suppress irrelevant information in the problem's text (Passolunghi & Siegel, 2001; Rivella et al., 2021) or to inhibit an overlearned procedure in favour of a new one (Khng & Lee, 2009; Viterbori et al., 2017). This strong relationship is also well established in the realm of literacy abilities. The role of WM was demonstrated in the acquisition of letters and words prior to school (Fuhs et al., 2014) and in reading comprehension among older students (Carretti et al., 2009, provide a review and meta‐analysis). Initial findings indicate that interference suppression plays a crucial role in the development of both phonological awareness and early orthographic knowledge in young children (Gandolfi et al., 2021; Shaul & Schwartz, 2014). These insights suggest a direct influence of EF on diverse aspects of school achievement, highlighting the multifaceted role of EF in supporting academic success across learning domains and developmental stages.

### Learning‐related behaviour and school achievement

LRBs can be seen as the behavioural expression of SRL, a broader construct encompassing metacognitive, motivational, and behavioural regulation during learning (Zimmerman, 2000). SRL involves planning, monitoring, and evaluating one's own learning processes, as well as managing motivation and emotional responses to challenges, enabling students to direct their learning in goal‐oriented ways. Several studies investigated the link between SRL, EF, and academic achievement, with studies indicating that SRL mediates the connection between EF and academic achievement (Grüneisen et al., 2023). While SRL provides the overarching framework, the present study focuses specifically on LRB, capturing the observable, classroom‐based manifestations of self‐regulation in first graders.

Typically assessed through teacher reports or classroom observations, LRBs provide insights into a young child's approach to the learning process rather than focusing on specific academic outcomes (McDermott et al., 2014). Several recent studies showed the crucial role of LRBs in the transition to primary school and academic achievement (Duncan et al., 2007; Hirvonen et al., 2020; Ponitz et al., 2009; Sánchez‐Pérez et al., 2018). Longitudinal studies show that LRBs in kindergarten predicts growth in math achievement from kindergarten to third grade (DiPerna et al., 2007) and literacy skills in fourth grade (Hirvonen et al., 2010, 2012).

### The mediating role of LRB in the EF‐school achievement association

Given the established results on the direct impact of both EFs and LRB on school achievement, and the increasing evidence of their critical role in promoting school success, a growing body of research explored the mediational hypothesis, positing that LRBs might act as mediators between EFs and academic achievement. That is, EFs impact school achievement both directly, supporting cognitive functions and information processing, and indirectly, promoting positive LRB which in turn supports academic achievement (Nesbitt et al., 2015). Nevertheless, results on this topic are mixed, with some studies finding a mediational role of LRB (Anthony & Ogg, 2020; Rutherford et al., 2018; Sasser et al., 2015) and others not (Brock et al., 2009). Another study found mediation when considering teacher‐reported grades but not when considering achievement test scores (Ponitz et al., 2009). In addition to the contrasting results, summarizing the existing literature (Table 1), some issues emerge. Despite the existence of some longitudinal studies, the mediational hypothesis remains understudied in younger students, despite EF and LRB having a crucial role in the transition to primary school. In addition, several studies analysed EF as a single factor, while only a limited number of them analysed the specific role of different EF domains. Moreover, when the multicomponential nature of EF was considered, each domain is measured with a single EF measure (Table 1). However, due to the impurity problem, that is a task designed to measure EF often measures also other cognitive functions and other EF components that were not intended to be measured, this practice is not recommended (Willoughby et al., 2014).

**TABLE 1:** Existing literature on the relationship between EF and LRB.

Study	Sample	EF (*n* task)	Achievement	Results
Anthony and Ogg (2020)	Kindergarten to fourth grade (longitudinal)	Cognitive flexibility (1) Working memory (1)	Science Math Reading	Mediational effect on both the EF domain and all the academic domains
Brock et al. (2009)	Kindergarten	Cool EF (1) Hot EF (1)	Math	No mediational effect
Heemskerk and Roebers (2023)	Second grade	Inhibition Shifting (1)	Language Math	Mediational effect on language for inhibition No effects for shifting
Nesbitt et al. (2015)	Prekindergarten	Unitary factor (5)	Literacy Language Math	Mediational effect on literacy and math
Neuenschwander et al. (2012)	Kindergarten to second grade (longitudinal)	Unitary factor (3)	Math Literacy	Mediational effect on school grade No mediational effect on standardized achievement test
O'Toole et al. (2018)	Preschool (5–6)	Cool EF (3) Hot EF (2)	Math Reading	Mediational effect on both school achievement only for WM
Rutherford et al. (2018)	Second to third grade (longitudinal)	Inhibition + cognitive flexibility (1) WM (1)	Math English/language arts (ELA)	Mediational effect of Inib/cf. for math grade and standardized tests No mediational effect for WM
Sasser et al. (2015)	Kindergarten to third grade	Unitary factor (3)	Math Reading	Mediational effect on reading skill No mediational effect on math
Sung and Wickrama (2018)	Kindergarten to first grade	Unitary factor (2)	Math Reading	Mediational effects for both the academic domains

Taken together, there is support for LRB as a mediator, but there remains a gap in our understanding of this relationship in younger students and the specific contribution of single EF and school achievement domains.

### The present study

The primary objective of this study was to enhance our understanding of the mediational hypothesis and to fill the existing gap in the literature. First, our study focused on first‐grade Italian children, recognizing that the transition from kindergarten to primary school represents a pivotal period marked by substantial changes in self‐regulation demand. Second, we considered the distinct contributions of the two EF components which are clearly distinguishable at the beginning of primary school, namely, inhibition and WM. Cognitive flexibility was not considered according to the existing literature about its later differentiation (Gandolfi et al., 2014). Each domain was assessed through two different tasks to account for the impurity problem. Third, both math and literacy attainment were considered in this study. This approach is informed by existing literature suggesting that inhibition and WM may be associated differently with reading, writing, and arithmetic. The study adopted a correlational design, and all analyses, including mediation, were conducted on concurrently collected measures.

Our hypothesis suggests that EF dimensions exert a positive influence on school achievement, operating both directly and indirectly through the mediation of LRBs. We hypothesize that better EF abilities are associated with higher achievement in early literacy and math and that LRBs play a mediational role between EF skills and achievement. LRBs include increased attention, active participation, heightened engagement in school activities, and greater independence in their work. No a priori hypothesis was established regarding potential variations in the mediational role of LRBs when considering different EF components.

## MATERIALS AND METHODS

### Participants

The research involved 98 students attending the first grade in a primary school in northern Italy. The school is in the city centre and is attended by families of middle socio‐economic status. The study was planned to be conducted in 2020; however, due to the COVID‐19 pandemic and the consequent closure of schools, it was interrupted, allowing the evaluation of only 58 students. It was then proposed to the first‐grade students in 2021, and another 40 students were evaluated. Before participation, parental consent was obtained for each student. The study adhered to ethical standards and guidelines set forth by the Italian Psychological Association and received approval from the local ethical committee at the University of Genoa, Italy. Participants who scored below the 5th percentile on the Coloured Progressive Matrices (Raven et al., 1998) or those without a complete assessment were excluded from the analysis. Consequently, the final sample comprised 91 participants, with 44 (48.4%) being male, and an average age of 6.62 years (SD = .3). No children were reported as having developmental disorders or disabilities. As a measure of socio‐economic status, maternal education was collected (Harding et al., 2015). Most mothers completed high school education (42%), while a smaller percentage completed only primary school (10%), middle school (27%), or university (21%).

### Procedure

The assessment was conducted during the second half of the school year (February–March) and involved four sessions, each lasting ~ 30 min. School achievement in math, reading, and writing was evaluated during two collective sessions in the classroom. EF were assessed in two individual sessions. EF assessments conducted in 2020 occurred in a quiet room at the school. However, in 2021, due to the pandemic situation, researchers were not permitted to assess children on‐site. Instead, evaluations were conducted remotely via computer while the children were at home, connected with the experimenters through a Skype video call. To facilitate remote evaluation, a PDF file containing task stimuli was created and presented to the children by the experimenter through screen sharing. In both cases, the assessment was carried out by trained psychology students. The four sessions were conducted ~ 1 or 2 days apart, so the assessment of each child was completed within 1 or 2 weeks. Additionally, teachers were requested to complete a questionnaire to assess LRBs at school.

### Measures

#### Fluid intelligence

Fluid intelligence was assessed using the Coloured Progressive Matrices Test (CPM; Raven et al., 1998). This test served as a control measure to ensure that children displayed typical cognitive abilities. It required completing a geometric figure by selecting the missing piece from six possible alternatives. The difficulty of the patterns increased progressively. The percentile corresponding to the number of correct answers (0–36) was coded.

#### Academic achievement

##### Math

For the evaluation of math skills, the Italian AC‐MT 6–11 (Cornoldi et al., 2012) was administered. Specifically, the collective task section was utilized, which included calculation (two additions and two subtractions), magnitude comparison (six comparisons), and reordering of numbers from the largest to the smallest and vice versa (eight comparisons in total). The analysis considered the number of correct responses ranging from 0 to 18. Test–retest reliability ranges from .68 to .77 depending on the task, whereas concurrent validity ranges from .50 to .58.

##### Reading

A silent reading task was administered to assess reading skills. In this task, children were required to identify, with a slash, the end of a series of 58 words written with no spacing between them (Bellocchi et al., 2014). The task had a time limit of 4 minutes, and the analysis utilized the number of correct responses, ranging from 0 to 58. Test–retest reliability is .80, whereas concurrent validity is .74.

##### Writing

To evaluate writing skills, a 40‐word dictation task was administered (Bellocchi et al., 2014). The task increased in difficulty, progressing from two‐syllable words to three‐syllable words. The analysis considered the number of errors, including incorrect or omitted words, with a range from 0 to 40. Test–retest reliability is .90 whereas concurrent validity is .78.

#### Executive function

##### Working memory

Backward digit span: in this task, the child is asked to recall a series of spoken numbers in the reverse order (Bisiacchi et al., 2005). Numbers are presented at a rate of one per second, with task difficulty increasing from two‐ to seven‐digit sequences. The task ends after three consecutive errors at the same sequence length. The score reflects the longest list length for which the child correctly recalls two sequences, ranging from 1 to 7.

Dual WM task: the task comprises 12 blocks of sentences; the number of sentences per block increases from 2 to 5. During this task, the child listens to each sentence and, at its conclusion, determines whether it is true or false. Furthermore, at the end of every block, the child is required to recall the last word of each sentence. One point is assigned for each correctly remembered word within a block, irrespective of the order (Desimoni et al., 2010). The task terminates if the child scores 0 in all three blocks of the same length. The score is calculated by summing up the points for correctly recalled words (range from 0 to 42). Reliability and validity measures are not provided by the manuals.

##### Inhibition

Matching familiar figures task (MFFT): the task involves selecting from a set of six alternative figures the one that matches the target picture positioned at the top of the page (Marzocchi et al., 2010). The task consists of 20 items. The recorded data includes the number of errors, ranging from 0 to 100, and the average time taken for the first response.

Inhibition – NEPSY‐II: the inhibition subtest from the Developmental Neuropsychological Assessment – Second Edition (NEPSY‐II; Korkman et al., 2007) was used. In this subtest, children are presented with a series of black and white shapes (squares and circles) or arrows (pointing up or down). Their task is to name either the shape or direction in the opposite manner, disregarding the colour (e.g., naming circles as squares and squares as circles). The number of errors, including self‐corrections, and the time taken for each response are registered. Additionally, a combined scaled score (*M* = 10, SD = 3) was also calculated according to the normative data. Internal reliability and test–retest reliability are .80 and .75, respectively.

#### Learning‐related behaviours

The Teacher Rating Scale for School Adjustment (TRSSA; Birch & Ladd, 1997; Ladd et al., 1999) was designed to assess various constructs reflecting the behavioural and relational adjustment of young children to school or classroom settings. In our study, we employed two specific subscales of the TRSSA. The first, Cooperative Participation (eight items, e.g., ‘Follows teacher's directions’ and ‘Uses classroom materials responsibly’), evaluates how children regulate their attention and behaviour in classroom activities complying with classroom rules and responsibilities. The second, Independent Participation (9 items, e.g., ‘Works independently’ and ‘Is tuned in to what's going on in the classroom’), reflects how children display independent or self‐directed behaviour in the classroom. Teachers provided ratings for each item, indicating whether it was ‘applicable’, ‘sometimes applicable’, or ‘not applicable’ to the child, with corresponding scores of 2, 1, and 0, respectively. A total score of LRBs was computed by summing up the scores from both scales, resulting in a range from 0 to 34 (Cronbach's = .91 and .92 respectively).

### Statistical analysis

The data analysis was performed using SPSS (version 25). Initially, differences between the two cohorts were investigated. A series of *t*‐tests for independent samples was performed to examine the mother's average level of education, as well as the fluid intelligence of the two groups. A *t*‐test was also executed to examine whether the mode of EF evaluation (in‐person vs. remotely) had an impact on children's performance. A Chi‐squared test was performed to investigate sex differences between the two groups. As the children attended the same school, we hypothesized to find no differences both in the descriptive characteristics and in the EF tasks. We anticipate comparable means between tasks administered in person in 2020 and the same tasks administered remotely in 2021.

Subsequently, a principal component analysis with promax rotation was undertaken on the EF measures. The objective was to confirm whether these tasks could load onto two factors, namely, inhibition and WM.

In the third step, correlations between measures were calculated to determine whether there were significant connections that could support the examination of mediational relationships.

Finally, the mediation hypothesis was assessed using the SPSS add‐on software PROCESS. This type of analysis verifies whether the relationship between two variables (EF dimension and learning skills) depends on another variable (LRBs). Regression analyses based on 5000 bootstrap samples were employed to determine path coefficients for the regression equations (Hayes, 2013). This analytical approach relies on estimating the indirect effect, which represents the overall mediation pathway. The indirect effect is defined as a product of the individual regression coefficients constituting the mediation pathway. The determination of significant mediation (indirect effect) is based on a 95% confidence interval that excludes zero. This holds true regardless of whether the independent variables exhibit a significant total effect on the dependent variable (Shrout & Bolger, 2002). In all models, sex, parents' education level, and Raven's scores were included as covariates to account for potential confounding factors.

## RESULTS

The comparison between the two groups of participants evaluated in 2020 and 2021 respectively revealed no differences in terms of socio‐economic status, fluid intelligence, or sex (all *p* > .05). As for the EF tasks performances of children evaluated in person versus remotely, no differences emerged in the accuracy indices. However, notable exceptions were observed in the time scores for both MFFT and Inhibition, leading to their exclusion from subsequent analyses (Table 2). Overall, the results suggest that particularly concerning the accuracy index, the assessment modality did not significantly influence children's performance. Consequently, we proceeded with the principal component analysis of the executive function (EF) measures. Specifically, we included the backward digit span, the dual WM task, MFFT‐errors, and the inhibition combined score. Results demonstrated that the backward digit span and the dual WM task loaded onto factor 1, termed WM, while MFFT‐Errors and the Inhibition combined score loaded onto factor 2, termed Inhibition (Table 3).

**TABLE 2:** Descriptive statistics and comparison between 2020 and 2021 samples.

	2020 (*n* = 54)	2021 (*n* = 37)	*t*	*p*
	*M* (SD)	*M* (SD)		
Span	2.87 (.73)	2.86 (.82)	.034	.973
Dual‐task	19.80 (6.41)	19.30 (6.15)	.371	.712
MFFT‐E	16.04 (9.45)	17.27 (9.95)	−.599	.551
MFFT‐T	15.63 (9.78)	20.50 (13.42)	−2.006	.048
Inhibition‐E	7.67 (5.36)	11.32 (11.65)	−1.785	.081
Inhibition‐T	106.78 (22.39)	117.95 (24.57)	−2.229	.028
Inhibition‐C	10.04 (2.53)	8.97 (2.61)	1.945	.055

*Note*: Inhibition‐E = inhibition subtest part B, errors; Inhibition‐T = inhibition subtest part B, time; Inhibition‐C = inhibition subtest part B, combined score; MFTT‐T = matching familiar figure test, time; MFTT‐E = matching familiar figure test, errors.

**TABLE 3:** Factor loadings for the principal component analysis concerning the EF tasks.

	WM	Inhibition
Span	.698	
Dual‐task	.894	
MFFT‐E		−.794
Inhibition‐C		.843

*Note*: Inhibition‐C = inhibition subtest part B, combined score; MFTT‐E = matching familiar figure test, errors.

Descriptive statistics and Pearson correlations are reported in Table 4.

**TABLE 4:** Descriptive statistics of school abilities and LRB and correlations among all measures.

	1	2	3	4	5	6
1. Inhibition	1					
2. WM	.359**	1				
3. Reading	.281**	.411**	1			
4. Writing	−.452**	−.362**	−.675**	1		
5. Math	.348**	.369**	.338**	−.380**	1	
6. LRB	.566**	.389**	.405**	−.503**	.395**	1
*M*			31.47	11.98	15.80	3.09
SD			18.20	10.34	3.09	7.01

** *p* < .01.

### Mediation model with working memory as a predictor

**FIGURE 1:**
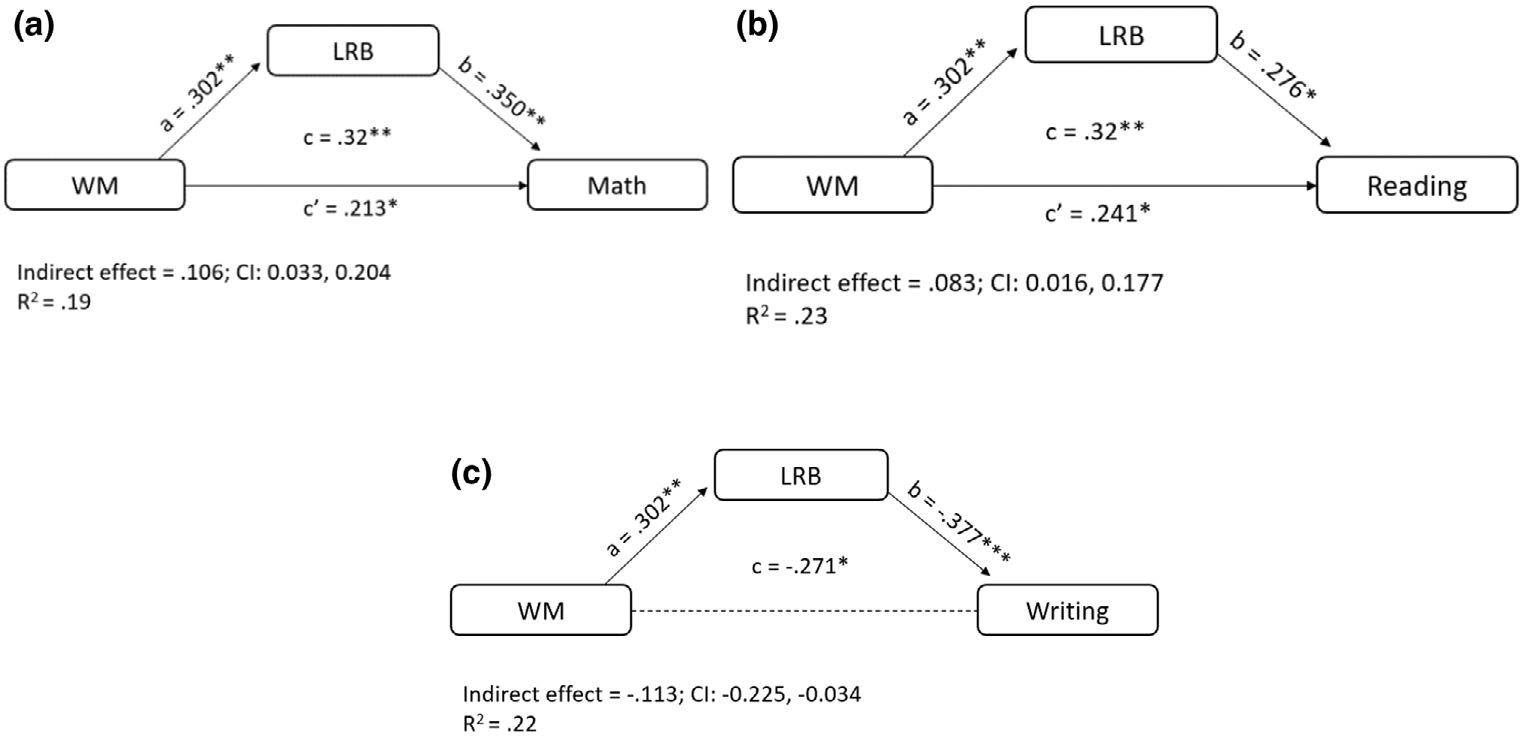
Mediational model with WM as a predictor and math (a), reading (b) and writing (c) as a dependent variable. Standardized effects are reported. Sex, parents' education level, and Raven's scores were included as covariates.

We conducted three mediational models, with the WM factorial score as the independent variable, LRB score as the mediator, and Math (Model 1a), Reading (Model 2a), or Writing (Model 3a) as the dependent variable. The results are depicted in Figure 1.

In Model 1a (Math), WM abilities predicted LRB, indicating that children with higher WM scores exhibited higher LRB scores (*p* = .003, CI [.760, 3.477]). The effect of LRB on the Math score was significant (*p* = .002, CI [.057, .252]), with higher LRB associated with higher Math scores. The mediation hypothesis was confirmed, as the indirect path was significant (CI [.033, .204]). The direct effect of WM on Math was also significant (*p* = .046, CI [.011, 1.306]), indicating partial mediation.

In Model 1b (Reading), similar to Model 1a, the effect of LRB on the Reading score was significant (*p* = .014, CI [.149, 1.284]). The mediation hypothesis was confirmed, with a significant indirect path (*β* = .083, CI [.016, .177]). The direct effect of WM on Reading was also significant (*p* = .023, CI [.613, 8.158]), indicating partial mediation.

In Model 1c (Writing), results indicated total mediation for Writing. The effect of LRB on the Writing score was significant (*p* < .001, CI [−.872, .240]), with higher LRB associated with lower Writing errors. The mediation hypothesis was confirmed, as the indirect path was significant (CI [−.225, −.034]). Unlike previous models, when LRB entered the model, the direct effect of WM on Writing was not significant (*β* = −.158, *p* = .126, CI [−3.732, .470]).

### Mediation model with inhibition as a predictor

**FIGURE 2:**
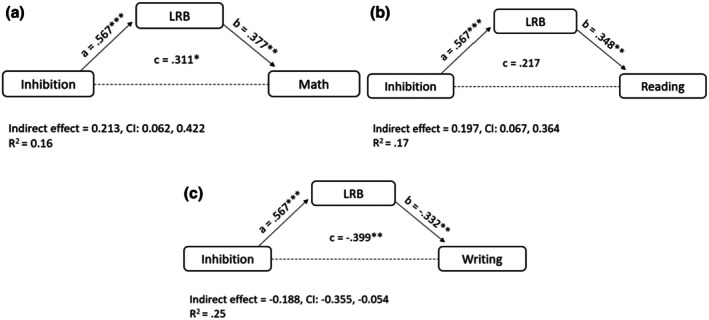
Mediational model with WM as a predictor and math (a), reading (b) and writing (c) as a dependent variable. Standardized effects are reported. Sex, parents' education level, and Raven's scores were included as covariates.

Similar to the WM models, three mediational models were conducted with the Inhibition factorial score as the independent variable, LRB score as the mediator, and Math (Model 2a), Reading (Model 2b), or Writing (Model 2c) as the dependent variable. Results are presented in Figure 2.

In Model 2a (Math), inhibition significantly predicted LRB, with higher Inhibition scores associated with higher LRB scores (*β* = .567, *p* < .001, CI [2.539, 5.406]). The effect of LRB on Math scores was significant (*p* = .004, CI [.056, .276]), with higher LRB associated with higher Math scores. The mediation hypothesis was confirmed, as the indirect path was significant (*β* = .213, CI [.062, .422]). Unlike the WM model (Model 1a), the direct effect of Inhibition on Math was not significant (*β* = .98, *p* = .481, CI [−.5479, 1.1532]), indicating total mediation.

In Model 2b (Reading), LRB significantly predicted Reading scores, with higher LRB associated with higher Reading (*p* = .006, CI [.257, 1.550]). The mediation hypothesis was confirmed, with a significant indirect path (*β* = .197, CI [.067, .364]). The direct effect of Inhibition on Reading was not significant (*β* = .194, *p* = .889, CI [−4.651, 5.359]), indicating total mediation.

In Model 2c (Writing), LRB significantly predicted Writing scores, with higher LRB associated with lower Writing errors (*p* = .007, CI [−.839, −.141]). The mediation hypothesis was confirmed, as the indirect path was significant (*β* = −.188, CI [−.355, −.054]). The direct effect of Inhibition on Writing was not significant (*β* = −.211, *p* = .113, CI [−4.877, −.523]), indicating total mediation.

In summary, the relationship between WM and school performance is partially mediated by LRBs (except for writing), indicating that WM affects learning skills both directly and indirectly through LRBs. In contrast, the relationship between inhibition and school performance is fully mediated by LRBs. This suggests that when LRBs are included in the models, inhibition is no longer associated with learning outcomes.

## DISCUSSION

The present study contributes to identifying factors that explain why individuals differ in their school performance when they enter primary school. This is crucial to promote children's school success as these results provide helpful insights to educators on how to support their students' skills or how to design school programmes.

EFs play a crucial role in supporting school achievement and fostering academic success (Cortés Pascual et al., 2019). Among various EF domains, WM is consistently identified as particularly relevant to school performance (Willoughby et al., 2012). In contrast, the evidence regarding the relationship between inhibition and academic performance is less conclusive. While some studies established a connection (e.g., Gilmore et al., 2015), others did not find a clear association (e.g., Waber et al., 2006). Moreover, research suggests that EFs, particularly WM, are primarily implicated in math achievement (Allan et al., 2014; Gerst et al., 2017), whereas the impact of EF on reading and writing tends to be relatively smaller (Gathercole et al., 2005, 2006; Swanson & Jerman, 2007). This nuanced perspective underscores the domain‐specific nature of the relationship between EF and academic outcomes and suggests that these associations may operate through both direct cognitive mechanisms and indirect pathways involving LRBs (Neuenschwander et al., 2012). However, this pathway has not yet been adequately explored in young students, nor has the specific contribution of distinct EF components to different domains of achievement been explored. Therefore, the present study aimed to extend current understanding about the mechanisms by which EF – specifically WM and inhibition – relate to academic achievement, by examining whether LRBs mediated such relationship.

Recognizing the variability in the impact of EF and LRBs across academic domains, as observed in previous research (Anthony & Ogg, 2020; Neuenschwander et al., 2012), we tested our mediational hypothesis in the context of math, reading, and writing skills. Our study aligns with prior investigations that have explored the mediational hypothesis (Nesbitt et al., 2015; Neuenschwander et al., 2012; Sasser et al., 2015). By extending these findings to the specific domains of math, reading, and writing skills and using two separate measures of inhibition and WM, this study contributes to a more nuanced understanding of the interplay between cognitive and behavioural mechanisms during the first year of primary school.

The study's findings indicate both direct and indirect relations via LRBs between WM and math and reading skills, whereas only an indirect relation emerged between WM and writing. These findings suggest that WM is related to academic outcomes not only through direct cognitive processes implicated in school tasks (e.g., maintaining number in mind during mental calculation; Bull & Scerif, 2001), but also through the association with LRBs (e.g., higher attention, independence, etc.; Blair, 2002). This mediating role of LRBs may help explain why previous studies have reported inconsistent or weak direct associations between WM and writing (Gathercole et al., 2005). In particular, for writing, our results indicate that WM may not contribute directly to performance; rather, it supports the development of behaviours that facilitate successful task completion and learning engagement (Alloway et al., 2009). As reported by Alloway et al. (2009), children with lower WM often exhibit classroom behaviours such as distractibility, forgetfulness, reduced attention span, and difficulties in remembering task instructions, which in turn are closely linked to lower academic outcomes, particularly in the writing domain.

As for the association between inhibition and school achievement, it emerged as fully mediated by LRBs, regardless of the academic domain considered (math, reading, or writing). This suggests that inhibition is primarily associated with academic performance indirectly via its relationship with LRBs. Inhibition emerges as a facilitator for optimal learning experiences, promoting appropriate behaviours in the school context and allowing children to fully benefit from their educational experiences. This is in line with previous research finding no association between inhibition and academic performance (Blair & Razza, 2007; Duncan et al., 2017; O'Toole et al., 2020).

These findings collectively indicate that the associations between EF and academic performance extend beyond their direct involvement in cognitive tasks and are consistent with theoretical models suggesting that EFs support academic achievement by enabling children to regulate attention, plan, and organize behaviour in learning contexts (Blair & Razza, 2007; Diamond, 2013). In other words, the results emphasize the importance of considering the behavioural aspects associated with EFs. Moreover, WM emerged as more consistently associated with learning outcomes than inhibition, aligning with prior research highlighting WM as a key process in complex learning tasks that require mental manipulation and sustained attention (Peng et al., 2016). Beyond theoretical implications, these findings have practical relevance. They suggest that early interventions designed to foster school readiness should not only strengthen EF skills but also promote LRBs, such as attention control, persistence, and cooperation. Supporting children in developing these behaviours may enhance their ability to translate EF capacities into successful learning experiences.

Other factors may contribute to our findings, particularly the age of the children and the tasks used to assess EFs. First, in our study, we included children in the first grade, recognizing the heightened influence of behavioural aspects on early academic outcomes. However, it can be hypothesized that the relationship between EFs, LRBs, and school achievement may change as children progress through the school years. LRBs may be more pertinent in the initial grades and potentially less significant in subsequent years. Second, the results related to inhibition could be influenced by the nature of this cognitive process and the specific tasks chosen for assessment. Inhibition is commonly categorized into response inhibition, involving the suppression of a behavioural response, and cognitive inhibition (interference control), responsible for directing attention towards relevant information while suppressing attention to non‐relevant information (Brydges et al., 2013). In our study, the tasks designed to evaluate inhibition primarily targeted the behavioural aspect, considered a key element of effective self‐regulation. Response inhibition encompasses the deliberate ability to withhold or adjust immediate responses, introducing a brief delay to better prepare for responding to environmental demands. This might explain the strong association between our measure of inhibition and the LRB measure, which assesses behaviour in the classroom, and why the direct impact of inhibition on school outcomes did not reach significance in the mediational model. The use of a measure specifically assessing interference control could have yielded different patterns of association. For example, Traverso et al. (2022) found that interference control, but not response inhibition, predicts early literacy skills in kindergarten children.

## LIMITATIONS AND FUTURE DIRECTIONS

Some limitations require consideration, and additional investigations are necessary to enhance our understanding of the link between EFs and academic achievement. The principal constraint in our study is the limited sample size, diminishing the statistical power of our analysis. Subsequent research should validate these findings with larger participant cohorts, allowing also more detailed analysis, as path models including all the EF domains together or latent model of mediation effects. Other confounding variables could also be considered, as for example vocabulary skills, language background (monolingual/bilingual), and preschool experience. Moreover, the mean score in the math tasks was relatively high (15 out of 18), suggesting that the task may have been relatively easy for the sample. However, this did not prevent us from detecting significant associations with EFs and LRBs. Nevertheless, future studies could consider using tasks with a wider range of difficulty to better capture individual differences in performance and potentially strengthen observed effects. Additionally, the inclusion of older children is essential to assess whether age and accumulated school experience may impact the identified relationships. Future research could also benefit from a longitudinal approach, investigating the developmental trajectory of EFs, LRBs, and their impact on academic outcomes across multiple school years. Lastly, incorporating diverse measures of inhibition, including those assessing interference control, can provide a more comprehensive understanding of how inhibition contributes to LRBs and academic success. Moreover, our assessment of LRBs captured only some aspects of self‐regulation in class. Future studies could include additional subscales or complementary measures to provide a more comprehensive view of children's LRBs.

### Implications

The study underscores the significance of both EFs and LRBs in shaping academic success during the first grade. These findings emphasize the necessity of implementing targeted interventions to address weaknesses in WM and inhibition, as well as providing adequate support for LRBs early in a child's school journey. Recent research highlights the effectiveness of universal classroom interventions designed to enhance EF and self‐regulation in all children (Diamond & Ling, 2020; Raver et al., 2011; Rivella et al., 2024). These types of approaches, aimed at all children in the classroom, could be particularly useful because teachers often struggle to identify children with poor EF, especially when such difficulties do not align with a clinical diagnosis, and to implement appropriate interventions. Concentrating on enhancing EF skills in the early stages of a child's academic path can yield long‐term benefits. It could enable children to develop the necessary skills for adapting to the school environment, fostering engagement in the learning process, and ultimately enhancing academic performance. These interventions may contribute not only to a more positive school climate but also to elevated academic achievement for all students.

## AUTHOR CONTRIBUTIONS

**Carlotta Rivella:** Investigation; writing – original draft; methodology; writing – review and editing; formal analysis; data curation. **Paola Viterbori:** Conceptualization; methodology; writing – review and editing.

## FUNDING INFORMATION

This work was supported by Liguria Region under the European Social Fund (ESF) Operational Programme 2014–2020, Axis 3 Education and Training.

## CONFLICT OF INTEREST STATEMENT

None.

## Data Availability

The data supporting this study are available from the corresponding author on request.
